
JOURNAL INFORMATION
==============================
NLM Title Abbreviation: Atlanta Med Surg J (1884)
NLM Journal ID: 101251623

Atlanta Medical and Surgical Journal (1884)

Publisher: Biomedical Journal Digitization Project

ARTICLE INFORMATION
==============================
PMCID: PMC9199295
PMID: 35829592
Article version: 1
Subjects: Society Reports

Atlanta Society of Medicine

Print publication date: 1898 Dec
Volume: 15
Volume Series: New Ser
Issue: 10
First page: 676
Last page: 678
License: This work is free of known copyright restrictions. For more information, please see PMC Back Issue Digitization.
License URL: https://creativecommons.org/publicdomain/mark/1.0/

==============================

### *SOCIETY REPORTS.*

---

### ATLANTA SOCIETY OF MEDICINE.

The Atlanta Society of Medicine held its regular meeting on the evening of November 3d, in the Society's rooms. The President, Dr. W. L. Champion, presided. The first paper of the evening was read by Dr. W. A. Crowe, on "Some Personal Views on the Management of Persistent Occipito-Posterior Presentations (published elsewhere).

Dr. V. O. Hardon opened the discussion. The speaker said that the subject here presented was a very important one, especially on two or three points. His views differed in some degree from those of the author of the paper. He agreed fully as to the importance and value of external palpation in determining the position of the fetus. In the large majority of cases, and only in those where the abdomen is covered by a large amount of adipose tissue, the diagnosis of presentation can always be made. Experience and education, however, are necessary in order to obtain the *tactus eruditus.* He examines the patient before and during labor. Yet we cannot confidently predict, even up to the ninth month, what will be the position when labor comes on, for the presentation sometimes changes. This was illustrated by a case recently had in practice. No man can always be sure as to position of the fetus, even with the best landmarks. We cannot always locate perfectly the fontanelles by digital examination, nor are the sutures always distinct. All of us are liable to error by digital examination, and hence the value of external palpation. This latter too is valuable in changing the position of the fetus if such be necessary. In regard to occipito-posterior positions, such produce long, tedious labors, twenty-four, thirty-six and even forty-eight hours in duration. Long labor is dangerous to child and to the mother, especially the second stage.

How can labor be hastened?

1. We may turn and deliver by breech. This increases the danger to both mother and child, for it is not easy of performance. His own experience is that forceps should be used just as soon as we find out that the occiput will not rotate to the front. Even when the head is high up in the pelvis we should use the forceps; using traction slowly upon the rods and aiding anterior rotation. The axis-traction forceps can be used with advantage. But when there is no tendency to rotate, then we will have a long, tedious labor with danger to both mother and child, and of rupturing the perineum; and even this latter can be limited, for such a rupture is better as compared with the danger to mother and child, by attempting version.

2. Laceration of the perineum. Men who do not have lacerations in their practice do not look for them. The great desideratum is to delay the head until we can obtain the shortest diameter for the passage—*i. e.*, the sub-occipito-bregmatic. You cannot support the perineum by pressure, and such a theory is erroneous.

3. Rectal expression, during the intervals of pain, is an excellent procedure, and one which the speaker frequently used. Laceration of the perineum is a comparatively light thing except, of course, when it is extensive and down into the bowel; and the speaker would not sacrifice mother and child for the sake of the perineum.

Dr. Kime. This is a very practical subject, and it would be strange if all doctors agreed as to the method of treatment. He agreed with the last speaker that to put the hand into the uterus for version is indeed a dangerous risk. It is all right when the manipulations are external, but when you carry your hand into the uterus and therefore a possible infection, the danger is increased. He preferred the forceps, even though there was more opportunity for rupturing the perineum. He did not believe in such a thing as "support to the perineum," and this was the view held by the best authorities. He believed that the effort to support was fruitless and frequently produced just the condition we wished to avoid. He believed in manipulating the child's head during the intervals of pain, and trying to adjust the head for the shortest diameter. He used the rectal expression.

Dr. Love. He believed in studying more closely external manipulation so as to become familiar with its use. He reported a case where external palpation showed its efficacy. He thought that physicians were too much afraid of the use of forceps. He believed in their more frequent use.

Dr. Amster. The speaker related a case showing where positions of the fetus sometimes change after a diagnosis has been made. On examining the case in the morning before labor had commenced he found a breech presentation, and when called at five o'clock the same afternoon, labor having commenced, he found the head presenting. He uses rectal expression, but not during the intervals. He uses it with the pain and retards too rapid progress of the child.

Dr. Gaston, Jr. The speaker had been pleased with the remarks of the evening. He believes that support of the perineum during labor is unnecessary. He believes that the passage of the shoulders is the most frequent cause of laceration. He believes also that long labors are frequently salutary in causing an adjustment of the parts. He would urge the necessity of retarding the rapid egress of the shoulders.

Dr. Hood. Speaker never has had a rupture from head but from shoulders of child. Finds no value from supporting the perineum. Has not had much difficulty in making out the presentation except in his first case, where he put his finger in the anus for the mouth.

Dr. Crowe (in closing) said he was glad to find so little difference between the views of the various speakers and himself.

Dr. Childs read a short paper on the history of the urethrascope and the cystoscope, and then presented a new cystoscope which he had originated and which had just been made for him by Tremann & Co.

---

***NOTICE.—This JOURNAL and THE INTERNATIONAL JOURNAL OF SURGERY one year for $2.00 cash.***
